# Vitronectin Expression in the Airways of Subjects with Asthma and Chronic Obstructive Pulmonary Disease

**DOI:** 10.1371/journal.pone.0119717

**Published:** 2015-03-13

**Authors:** Lina M. Salazar-Peláez, Thomas Abraham, Ana M. Herrera, Mario A. Correa, Jorge E. Ortega, Peter D. Paré, Chun Y. Seow

**Affiliations:** 1 School of Medicine, Universidad CES, Medellín, Colombia; 2 Penn State Microscopy Imaging Core, Penn State Hershey College of Medicine, Hershey, Pennsylvania, United States of America; 3 Instituto Nacional de Medicina Legal y Ciencias Forenses, Medellín, Colombia; 4 Clínica Cardio VID, Medellín, Colombia; 5 James Hogg Research Centre, University of British Columbia, Vancouver, BC, Canada; University of Pittsburgh, UNITED STATES

## Abstract

Vitronectin, a multifunctional glycoprotein, is involved in coagulation, inhibition of the formation of the membrane attack complex (MAC), cell adhesion and migration, wound healing, and tissue remodeling. The primary cellular source of vitronectin is hepatocytes; it is not known whether resident cells of airways produce vitronectin, even though the glycoprotein has been found in exhaled breath condensate and bronchoalveolar lavage from healthy subjects and patients with interstitial lung disease. It is also not known whether vitronectin expression is altered in subjects with asthma and COPD. In this study, bronchial tissue from 7 asthmatic, 10 COPD and 14 control subjects was obtained at autopsy and analyzed by immunohistochemistry to determine the percent area of submucosal glands occupied by vitronectin. In a separate set of experiments, quantitative colocalization analysis was performed on tracheobronchial tissue sections obtained from donor lungs (6 asthmatics, 4 COPD and 7 controls). Vitronectin RNA and protein expressions in bronchial surface epithelium were examined in 12 subjects who undertook diagnostic bronchoscopy. Vitronectin was found in the tracheobronchial epithelium from asthmatic, COPD, and control subjects, although its expression was significantly lower in the asthmatic group. Colocalization analysis of 3D confocal images indicates that vitronectin is expressed in the glandular serous epithelial cells and in respiratory surface epithelial cells other than goblet cells. Expression of the 65-kDa vitronectin isoform was lower in bronchial surface epithelium from the diseased subjects. The cause for the decreased vitronectin expression in asthma is not clear, however, the reduced concentration of vitronectin in the epithelial/submucosal layer of airways may be linked to airway remodeling.

## Introduction

Vitronectin, a glycoprotein encoded by the *VTN* gene, is a cell adhesion factor found in plasma and extracellular matrix (ECM) [1–5]. It is involved in diverse biological processes including regulation of coagulation pathways and formation of the membrane attack complex (MAC), cell attachment and migration, wound healing and tissue remodeling [6–9]. Vitronectin has been identified as a marker of profibrotic activity in several tissues, such as liver, heart and kidney [10–14]. It also has antimicrobial properties through its heparin-binding domains [15,16]. The inhibitory effect of vitronectin on MAC formation is used by host cells to minimize MAC-mediated self-reactivity during microbial infection [6]. Some gram-negative bacteria utilize this inhibitory effect to evade MAC deposition on their cell surface [17–19]; while gram-positive bacteria and some fungi utilize vitronectin as a cross-linker to adhere to host cells to promote adhesion and internalization mediated by host cell [19–22].

In the respiratory system, vitronectin has been found in exhaled breath condensate and bronchoalveolar lavage fluid of control individuals and subjects with interstitial lung disease [23–27]. It has been shown that vitronectin is synthesized by alveolar macrophages *in vitro* and it has been suggested that this is one of the sources of vitronectin found in the lumen of the respiratory tract [25,28]. Whether the level of vitronectin expression is altered in chronic lung diseases such as asthma and chronic obstructive pulmonary disease (COPD) is not known.

Asthma and COPD are respiratory diseases characterized by various degrees of airflow obstruction associated with chronic inflammation and tissue remodeling [29,30]. Pathological changes in asthma are confined primarily to the conducting airways, while in COPD both the conducting airways and the lung parenchyma are affected [29,30]. The clinical history of asthma and COPD includes episodes of acute exacerbations, chronic inflammation, airway obstruction and hyperresponsiveness [31]. The exacerbations are often triggered by respiratory viral infections, as well as by bacterial colonization of the respiratory tract [32–36]. The cellular and molecular mechanisms of exacerbations are still poorly understood [37]. Because of its complex roles in immune cell recruitment and subsequent inflammation, the inhibition of self-reactivity of the innate immune response, antimicrobial properties, and promotion of microbial colonization and internalization, vitronectin may be an important player in modulating the frequency of exacerbations in asthma and COPD. The purposes of this study are to identify possible cellular sources of vitronectin in the respiratory tract and to determine if there is differential expression in individuals with asthma and COPD. The finding will help in determining whether cells in the respiratory track contribute to the accumulation of vitronectin in the airways and whether the abundance of vitronectin is altered in asthma and COPD.

## Materials and Methods

### Tissue samples

Bronchial tissue from 7 asthmatic, 10 COPD, and 14 control subjects (with no known trauma to the lung as the cause of death), were obtained during regular autopsy by forensic pathologists from the National Institute of Legal Medicine and Forensic Sciences of Colombia, and used for immunohistochemical analysis. All subjects were over 18 years of age at the time of death (S1 Table). In a separate set of experiments, human bronchial tissues (6 asthmatics, 4 COPD and 7 controls) were obtained from lungs donated for medical research with written informed consent through the International Institute for the Advancement of Medicine (IIAM: Edison, NJ- http://www.iiam.org/) and James Hogg Research Centre Biobank (S2 Table). These samples were used for immunofluorescence, 3D confocal imaging and quantitative colocalization analysis. The study was approved by the University of British Columbia—Providence Health Care Ethics Committee (Certificate #H06–03267), and the Ethics Committee of the Universidad CES (Project Code 127, Act 44/November 22, 2011).

To demonstrate the presence of vitronectin RNA and protein in bronchial surface epithelium, brushings from 12 patients who underwent diagnostic bronchoscopy at Clinica Cardio VID (Pulmonology Service, Medellin, Colombia) were sampled (S3 Table) with written informed consent from patients. Approval by the Ethics Committee of the Universidad CES was obtained for this part of the study (Project Code 127, Act 44/November 22, 2011). Bronchoscopy procedures are described in full detail in S1 Methods.

### Tissue preparation

Samples were fixed in 10% buffered formalin for subsequent pathological examination and immunohistochemistry/immunofluorescence procedures. Sections of 5–7 μm thickness were obtained from paraffin-embedded tissue blocks, stained with hematoxylin-eosin (H&E), and examined to classify samples as healthy control (HC) or diseased. Histopathological diagnosis of possible asthma and COPD were in accordance with Husain´s criteria for those obtained by autopsy [38] and confirmed by certified pathologists. Additional sections were placed on poly-L-lysine-coated slides (Thermo Fisher Scientific; Suwanee, GA, USA) for immunohistochemistry and immunofluorescence analysis. For the lungs from IIAM, the same asthmatic lungs (physician diagnosed) used in the present study were also examined for evidence of airway remodeling in a previous study that found significantly thicker airway wall in the asthma group compared with the non-asthma group [39].

### Antibodies

For immunohistochemistry, mouse monoclonal anti-human vitronectin antibody (clone VIT-2, isotype IgM, code V7881) (Sigma-Aldrich; St. Louis, MO, USA) was used as primary antibody. As secondary antibody, an anti-mouse IgM peroxidase conjugated antibody produced in goat (μ-chain specific, code A8786) (Sigma-Aldrich; St. Louis, MO, USA) was used.

For immunofluorescence the following primary antibodies were used: mouse monoclonal antibody against the alpha-1 subunit of sodium/potassium ATPase (clone 464.6, isotype IgG1, code ab7671), mouse monoclonal anti-lactoferrin antibody (clone 2B8, isotype IgG1, code ab10110), mouse monoclonal anti-MUC5AC antibody (clone 45M1, isotype IgG, code ab3649), and rabbit monoclonal anti-vitronectin (clone EP873Y, isotype IgG, code ab45139), all from Abcam (Cambridge, MA, USA); as well as mouse monoclonal anti-human MUC5B (clone 8C11, isotype IgG2a, code SAB1403512) from Sigma-Aldrich (St. Louis, MO, USA). Alexa Fluor 488 goat anti-mouse IgG (code A11029) and Alexa Fluor 594 goat anti-rabbit IgG (code A11037) from Life Technologies (Grand Island, NY, USA) were used as secondary antibodies. For more detailed description of antibodies, see S4 Table.

### Immunohistochemical staining

Immunohistochemical staining methods for bright-field histological imaging are described in full detail in S1 Methods.

### Morphological and stereological measurements

Initial histological verification of the cell type marked by anti-vitronectin antibody was performed with the periodic acid-Schiff (PAS) assay, a method for mucous cell staining. Micrographs were superimposed on immunohistochemical images, using Fiji software [40] (S1 Fig).

The immunohistochemical sections were examined with a light microscope (Nikon Eclipse E200) (Nikon Inc.) at magnification from x100 to x400. 151 digital images (average of 5 per subject) were acquired by using a digital camera (Nikon Digital Sight DS-5M). Each digital image was taken from different microscopic fields visualized with x10 objective. The submucosal gland area in airway tissue and the area fraction of the gland occupied by vitronectin staining were measured with the image analysis software, Lucia version 5.3 (Laboratory Imaging; Praha; Czech Republic). For stereological analysis, an 81-point grid was overlaid on each image (average of 5 per subject, total of 151 images); the Cavalieri principle was applied to estimate volume fractions [41] Points hitting glandular tissue in the bronchial tissue indicated volume fraction of submucosal glands referenced to bronchial tissue volume (Gvf), and those on sites of vitronectin expression in submucosal gland indicated volume fraction of vitronectin-expressing cells relative to total gland volume (Vvf). Medians of glandular surface area, percentage of glandular area occupied by vitronectin, Gvf and Vvf, were measured in each subject and averaged for each subject group.

### Immunofluorescence staining

The immunofluorescence staining methods are described in full detail in S1 Methods.

### 3D confocal microscopy

Confocal images of fluorescence labeled sections were acquired with a Leica AOBS SP2 laser scanning confocal microscope (Leica, Heidelberg, Germany), using a high resolution Leica 63×/1.4 plan apochromatic oil immersion objective. The acquisition software was Leica Confocal Software TCS SP2. The laser lines used were 405 nm (for DAPI), 488 nm (for Alexa Fluor 488) and 594 nm (for Alexa Fluor 594), produced by ultraviolet diode, argon and helium-neon lasers (Leica AOBS SP2 module), respectively. The respective emission signals were collected sequentially to avoid cross excitation, as well as the crosstalk using AOBS tunable filters as follow; 410–480 nm for DAPI, 504–571 nm for Alexa Fluor 488, and 597–751 nm for Alexa Fluor 594. While collecting the images, appropriate PMT offset level was used to minimize the auto-fluorescence and images were framed an average of 3–4 times to minimize the noise. All images and spectral data were captured using the PMT detectors (R6357, Hamamatsu) located inside the scan head. Spectral scanning was performed throughout the experiments to confirm the specificity of the Alexa Fluor signal emission profiles.

### Voxel quantification

Volocity 3D Image Analysis Software (PerkinElmer; Waltham, MA, USA) was used to collate image stacks (80–160x1024x1024 pixels) and to quantify voxel volume and voxel intensities. A voxel is the 3-dimensional equivalent of a pixel, the smallest discrete spatial component of a digital volume. Volumes within Volocity consist of voxels, each with an associated color and /or intensity value. For the analysis, the lower threshold level in the histogram was set to the second standard deviation of the mean voxel intensity value to exclude all possible background voxel values. The sum of all voxels and voxel intensities above this threshold value was determined.

For 3D image data set acquisition, the excitation beam was first focused at the maximal signal intensity focal position within the lung tissue sample and the appropriate PMT gain level was then selected to obtain the pixel intensities within range of 0–255 (8-bit images) using a color gradient function. Later on, the beginning and end of the 3D stack (i.e. the top and the bottom optical sections) were set based on the signal level degradation. Series of 2D images for a selected 3D stack volume were then acquired with 1024 x 1024 pixels and were line averaged 3–4 times depending on the noise level. The 3D stack images with optical section thickness (z-axis) of approximately 0.3 μm were captured from tissue volumes. For each tissue volume reported here, z-section images were compiled and finally the 3-dimensional image restoration was performed.

Volocity software also was used for quantitative colocalization analysis. Pearson’s correlation coefficient (R_p_) and Mander’s colocalization coefficients (M_x_ and M_y_) were calculated. The most commonly used quantitative estimate of colocalization is the Pearson’s correlation coefficient (R_p_), which is applied to measure colocalization within dual color fluorescence images [42]. The colocalization coefficient characterizes the degree of overlap between two channels in a microscopy image. R_p_ is a measure of the covariance between two signals, which depends on the amount of colocalized signals in both channels, and is given by:

$$R_{p}=\frac{\sum_{i}\left(G_{i}-G_{A})(R_{i}-R_{A}\right)}{\sqrt{\sum_{i}{\left(G_{i}-G_{A}\right)}^{2}\times \sum_{i}{\left(R_{i}-R_{A}\right)}^{2}}}$$

where G_i_ and R_i_ are the fluorescence intensities of the green and red emissions respectively. G_A_ and R_A_ are the average intensities of the green and red emissions respectively. R_p_ is a robust colocalization quantity and is unaffected by the detector settings such as gain and offset levels of individual channels, but strongly affected by the image noise. A positive number for the R_p_ reflects the degree of overlap of fluorescent signals of molecular species with 1.0 representing a complete overlap, while a value of zero indicates the random overlap or placement. A more biologically meaningful set of coefficients are the Mander’s colocalization coefficients, M_G_ and M_R_. For a particular channel, the ratio of the colocalized voxel intensities (G_colocalized_ or R_colocalized_) to the total voxel intensities (G_i_ or R_i_), define the interacting fraction for that particular molecular species.

$$M_{G}=\frac{\sum_{i}G_{colocalized}}{\sum_{i}G_{i}},M_{R}=\frac{\sum_{i}R_{colocalized}}{\sum_{i}R_{i}}$$

By definition, M_G_ and M_R_ are unaffected by the detector gain level, but affected by the offset level and the noise.

To compare vitronectin expression between subjects, an average of five 2D images per tissue section were randomly selected. Then, vitronectin pixel intensity per visual field was estimated and normalized by the epithelial area. In addition, the observer was blinded with respect to the subjects’ diagnosis.

### NHBE and NCL-H23 culture

As bronchial brushings could contain some minute quantity of blood cells [43], Clonetics Normal Human Bronchial/Tracheal Epithelial cells (NHBE, Lonza, Walkersville, MD), an in vitro model of human bronchial epithelium [44–47], as well as NCL-H23 cells (from adenocarcinoma of epithelial origin, American Type Culture Collection, Manassas, VA, USA), were therefore used to show that cultured bronchial epithelial cells retain the ability to express vitronectin (mRNA). Approval by the Ethics Committee of the Universidad CES was obtained for this part of the study (Project Code 206, Act of May 14, 2013).

Primary NHBE cells, obtained from a 40-years-old female donor (lot number 307177), (>5 x 10^5^ cells/mL), were seeded (passage 2) into cell culture flasks T-75, containing bronchial epithelial basal medium (BEBM) supplemented with growth factors provided in the SingleQuot kits (Lonza, Walkersville, MD, USA). Once sub-confluence (65–75%) was achieved, the cells were submitted to RNA extraction. NCL-H23 cells were grown in flasks T-75, in Roswell Park Memorial Institute (RPMI)-1640 (Sigma-Aldrich, St Louis, MO, USA) supplemented with 10% heat inactivated fetal bovine serum (Lonza-BioWhittaker, Walkersville, MD, USA), until 70% of confluence. All the cells were cultured in triplicate using a humidified 5% CO_2_ incubator at 37°C.

### RNA analysis

The cells stored in RNAlater were recovered by vortexing and centrifugation. The total RNA was isolated using AllPrep DNA/RNA/Protein kit (Qiagen, Valencia, CA, USA), according to the supplier´s instructions. Each RNA extraction assay was accompanied by a sham extraction, using all reagents but with no cells present, as a negative control or “non-template control”. Total RNA was eluted in 30 μL free-RNAses water. The concentration and purity of RNA was determined using a NanoDrop 1000 spectrophotometer (NanoDrop Technologies, Wilmington, DE, USA). The yield of RNA ranged from 7.9 to 127.6 ng/μL for bronchial brushings, and 100 to 200 ng/μL for cellular cultures. The absorbance ratio at 260/280 nm of the total RNA extracted from bronchial specimens ranged from 1.85 to 1.98, and 2.04 to 2.06 for NHBE and NCL-H23 cultures respectively. Total RNA was stored at -80°C until use.

Double stranded cDNA was synthesized from 80 ng of total RNA per reaction, using QuantiTec Reverse Transcription (Qiagen, Valencia, CA, USA), as per the manufacturer’s recommendations. This kit contains a genomic DNA wipeout buffer to eliminate genomic DNA contamination from starting RNA samples. However, a “RT-control” (no reverse-transcriptase control) was included to detect the presence of amplification products derived from genomic DNA. A C1000TM Thermal Cycler (Bio-Rad, Hercules, CA, USA) was used for RT-PCR reactions.

Messenger RNA sequences for vitronectin and glyceraldehyde 3-phosphate dehydrogenase (GADPH) were obtained from the Genbank database. GADPH was selected as the house-keeping gene amongst five other genes (β-actin, RPL32, PSMB2, PPIA, RPL13A), as it showed the lowest standard deviation for the evaluated samples (data no shown). Primers were designed to span exon—intron boundaries so as to prevent amplification of any genomic DNA, using Primer3 software [48]. The chosen primer sequences were checked for specificity against the Genbank database. The primer sequences, melting temperatures and sizes of the PCR products are shown in S5 Table.

The reactions of quantitative real time-Polymerase Chain Reactions (qPCR) were performed using QuantiTect SYBR Green PCR kit (Qiagen, Valencia, CA, USA), according to the manufacturer’s instructions, but with minor modifications. Briefly, 1 μL cDNA working solution was used in a final qPCR reaction volume of 25 μL, containing 12.5 μL master mix and 0.4 pM of each primer. The conditions for the PCR included a step of initial heat activation during 15 min at 95°C, followed by 40 cycles of PCR (94°C for 15 seconds, 62°C for 15 seconds and 72°C for 10 seconds) and final extension was 72°C for 5 minutes. A melting curve was constructed by heating from 65°C to 95°C with temperature steps of 0.4°C to confirm that a single specific product was generated. All reactions were performed in triplicate using a Corbett Rotor-Gene® 6000 (Qiagen, Valencia, CA, USA). Negative controls (PCR mix without cDNA or PCR mix with RT-PCR control) were included at the pre-test stage to exclude false positive results. Amplification efficiency for each reaction was calculated using LinRegPCR program (2012.0) [49]. The average efficiency for each gene was calculated, being 86.07 ± 4.82 for GADPH, and 87.23 ± 3.88 for *VTN* gene.

To verify the band size of amplicons, q-PCR products were separated by running through 1.5% agarose gel and staining with GelGreen (Biotium Inc. Hayward, CA, USA). Relative expression of VTN transcript was calculated using REST (Relative Expression Software Tool) 2009 software [50]. Bronchial brushings with normal histological and microbial findings were used as controls.

### Western-blot analysis

Western-blot analysis was used to quantitatively assess vitronectin expression in bronchial brushing samples. Proteins were extracted from bronchial epithelial cells using AllPrep DNA/RNA/Protein kit (Qiagen, Valencia, CA, USA), following the manufacturer’s instructions. Protein concentrations were assessed using a bicinchoninic acid (BCA) protein concentration kit (Sigma-Aldrich; St. Louis, MO, USA). Details of western-blot procedures are reported in S1 Methods.

### Statistical analysis

Normal distribution of variables was assessed with D´Agostino-Pearson test. Kruskal-Wallis H Test, with Dunn´s multiple comparison post test, was used to compare bronchial expression of vitronectin between groups, as well as to establish differences in colocalization analysis. Student's t test was performed to establish differences in expression of the 65- and 75-kDa glycoprotein isoforms in bronchial surface epithelium between controls and diseased subjects. Statistical significance was set at P<0.05. GraphPad Prism 6.0 (GraphPad Software, Inc., La Jolla, CA, USA) was used as statistical software.

## Results

### Vitronectin expression in bronchial submucosal glands

**Fig 1:**
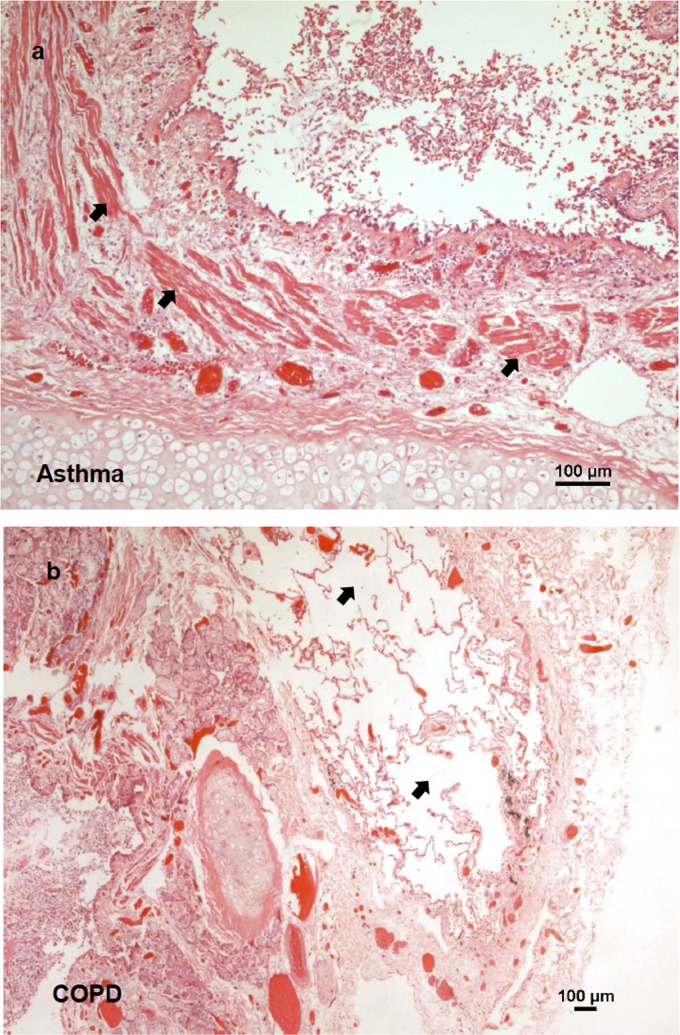
Lung structural change in asthma and COPD. **a**) Haematoxylin and eosin staining of bronchial tissues from asthmatic subjects. Arrows point to thickened smooth muscle cell bundles. **b**) Same staining as in panel **a**, for tissues from COPD subjects. Arrows point to emphysematous alveolus space.

Bronchial tissue from a total of 31 subjects: 7 asthmatics, 10 COPD and 14 HC were used for morphological analysis and to assess vitronectin expression. Morphological analysis with H&E of lung tissue from individuals classified as HC showed normal morphology and the lack of inflammatory cells in airway submucosa or signs of tissue remodeling. Bronchial sections from subjects classified as asthma exhibited overall thickening of the airway wall and increased smooth muscle layer (Fig. 1a). COPD individuals exhibited squamous metaplasia, mononuclear cell infiltration, and enlarged alveoli (Fig. 1b).

Stereological analysis of a total of 151 images (average of five per subject) was performed to evaluate vitronectin expression among different groups. Median values of measurements were obtained in each subject and in the total sample. Comparisons among the different groups are summarized in Table 1.

**Table 1:** Stereological measurements of cadaveric bronchial tissues according to histopathological diagnosis.

Condition	Healthy controls (n = 14)	Asthmatic (n = 7)	COPD (n = 10)	P
**Age (years)**	28.5 (24.5–38.0)	33.0 (24.0–38.0)	60.5 (43.8–72.3)	0.0003^a^
**Sex** ^b^: **Male**	13 (92.9%)	6 (85.7%)	8 (80.0%)	0.646
**Glandular area (mm^2^)**	0.29 (0.25–0.41)	0.29 (0.22–0.47)	0.30 (0.23–0.51)	0.8678
**Area occupied by vitronectin in SMG (percentage)**	17.30 (10.87–20.46)	9.70 (6.01–13.28)	5.17 (2.73–8.06)	0.0010 ^a^
**Glandular volume fraction**	0.39 (0.30–0.48)	0.35 (0.28–0.54)	0.40 (0.28–0.55)	0.9566
**Vitronectin volume fraction**	0.29 (0.21–0.40)	0.07 (0.03–0.08)	0.04 (0.03–0.07)	< 0.001 ^a^

Quantitative variables are expressed as medians (interquartile range)

^a^ Statistically significant values by Kruskall-Wallis H test.

^b^ Values expressed as absolute and relative (in parenthesis) frequencies.

**Fig 2:**
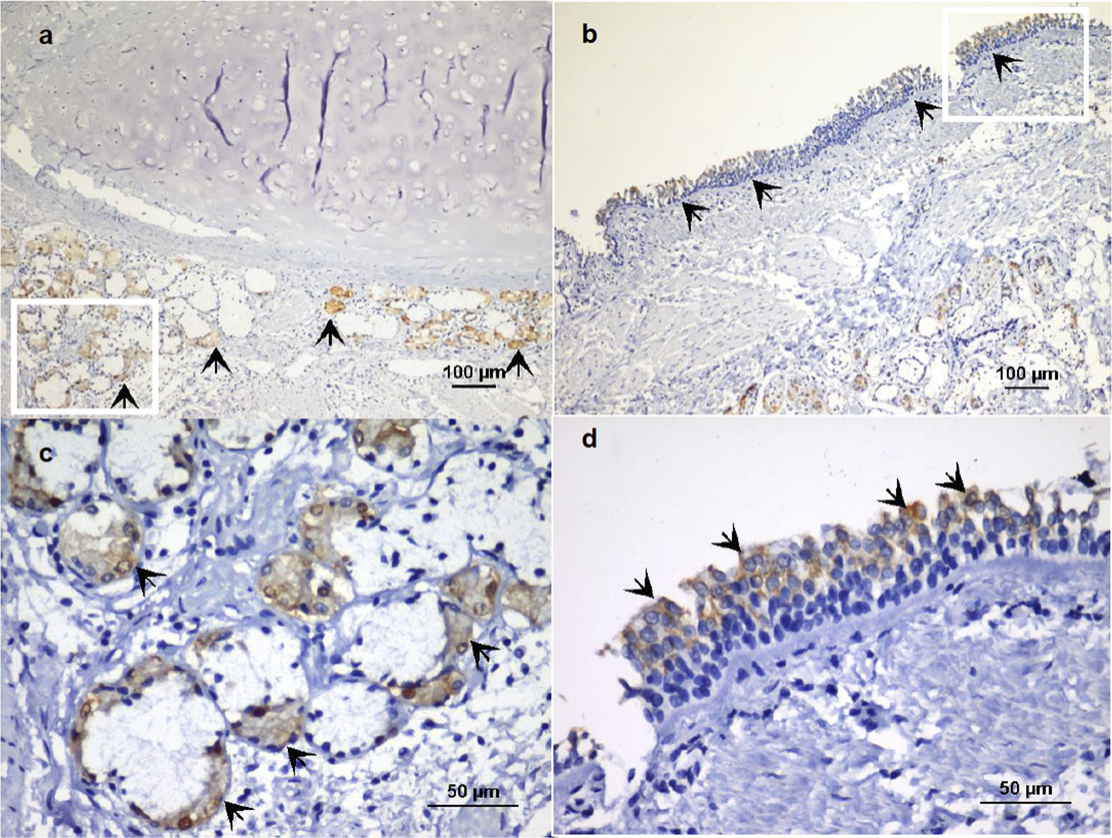
Localization of vitronectin in human bronchial epithelium. Indirect immunoperoxidase staining of paraffin sections from cadaver tissue (healthy control). **a**) Vitronectin expression in submucosal glands of a mainstem bronchus (arrows), magnification x100. **b**). Presence of vitronectin in the bronchial surface epithelium (arrows), magnification x100. **c-d**). Enlarged sections from panels **a** and **b**, magnification x400.

**Fig 3:**
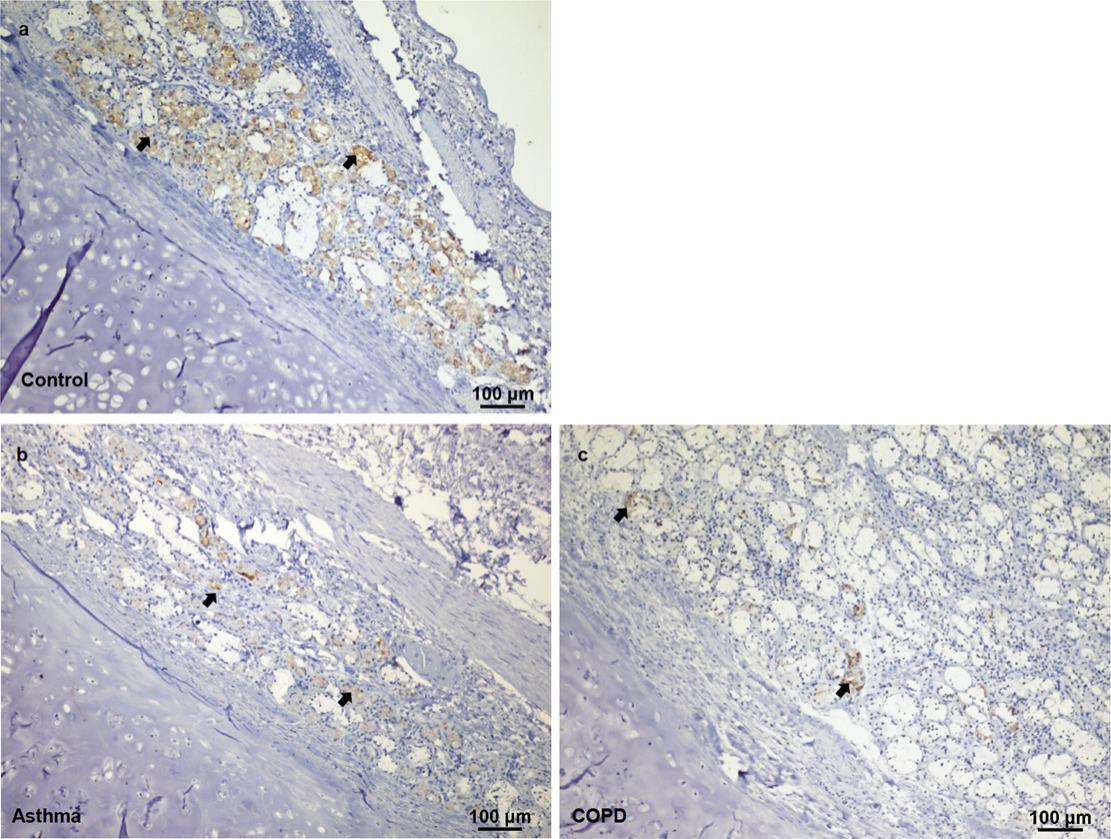
Vitronectin expression in healthy, asthmatic, and COPD individuals. Immunohistochemical staining shows vitronectin expression in airway tissues (arrows) from the control (**a**), asthmatic (**b**) and COPD (**c**) individuals.

The area occupied by vitronectin-positive pixels in the submucosal glands (Fig. 2a and 2c) was confined to serous cells (acini), as demonstrated by superimposition of PAS micrographs on immunohistochemical images (S1 Fig). Vitronectin was present in both HC and diseased subjects. However, the percent area of vitronectin expression was significantly lower in asthma and COPD subjects (p = 0.0010) (Fig. 3) (Table 1). A Dunn´s post test showed there was a difference between HC and COPD subjects (p<0.001). There was no difference in the glandular area or glandular volume fraction (Gvf) in submucosal tissues between the two groups. However, the volume fraction of the vitronectin stained tissue (Vvf) was significantly lower in asthmatic and COPD subjects (p<0.001). The post hoc test showed differences between asthmatic and HC (p = 0.01), as well as between COPD individuals and HC (p<0.001) (Table 1). Besides the submucosal glands, vitronectin expression was also observed in the surface epithelium, especially in the apical zone (Fig. 2b and 2d).

### Vitronectin expression by serous epithelial cells in submucosal glands

In order to identify the epithelial cell type expressing vitronectin in bronchial submucosal glands (serous or mucous epithelial cells), we performed a quantitative colocalization analysis on 3D confocal fluorescence images. A double-staining technique was used for simultaneous labeling of lactoferrin (a marker of serous cells) or MUC5B (a marker of mucous gland cell), and vitronectin. Airway tissues from a total of 17 donor lungs (6 asthmatics, 4 COPD and 7 HC) were used.

**Fig 4:**
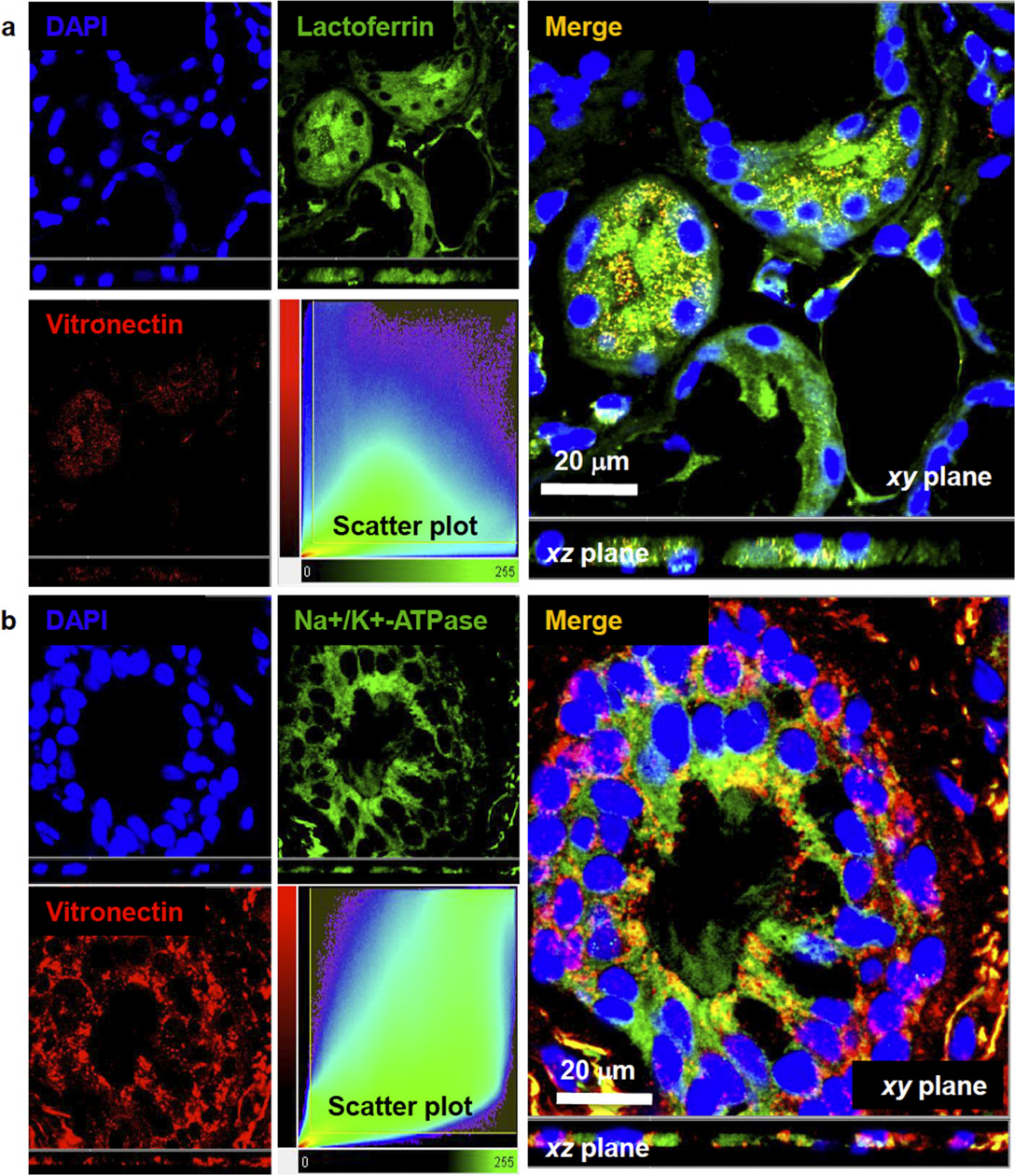
Colocalization of vitronectin and lactoferrin or alpha-1 sodium/potassium ATPase (Na^+^/K^+^-ATPase). **a)** Confocal images showing localization of lactoferrin, a marker of serous epithelial cells (green fluorescence), and vitronectin (red fluorescence) in bronchial submucosal glands. Overlay of the green and red channel shows that there is colocalization between lactoferrin and vitronectin, and indicates that vitronectin is inside serous cells in the airway submucosal glands (R_p_ 0.55, IQR 0.47–0.67). **b)** Confocal images showing localization of Na^+^/K^+^-ATPase (green fluorescence), a marker of cell membrane, and vitronectin (red fluorescence) in a collecting duct of a bronchial gland. Pearson´s correlation coefficient (0.61, IQR 0.54–0.71) shows not only that vitronectin is inside the bronchial epithelial cells, but also is associated with the cell membrane. Nuclei were counterstained with DAPI (blue fluorescence). *xy* and xz views are shown to provide sufficient spatial distribution details in the 3D subcellular space. The colocalization maps (scatter plots) of voxels are shown. Lactoferrin and Na+/K+-ATPase emission intensities are plotted on the x-axis, while vitronectin is on the y-axis. R_p_, Pearson´s colocalization coefficient; IQR, interquartile range.

Dual labeling experiments with lactoferrin and vitronectin demonstrated that vitronectin is expressed by serous epithelial cells in submucosal glands of the airway tree, as shown in Fig. 4a. In the total sample, the median value of Pearson’s correlation coefficient was 0.55 (IQR 0.47–0.67), indicating a partial interaction between both markers at the subcellular level. Median values for Mander’s colocalization coefficients (M_G_ and M_R_) for lactoferrin and vitronectin were 0.74 (IQR 0.60–0.79) and 0.93 (IQR 0.89–0.94), respectively. These results suggest that most of the vitronectin molecules are located near lactoferrin molecules. Intracellular presence of vitronectin in serous epithelial cells of bronchial submucosal glands was confirmed by the quantitative colocalization analysis for alpha-1 sodium/potassium ATPase, a marker of cell membrane, and vitronectin (Fig. 4b). Vitronectin was shown to be present inside the serous cells, and more specifically, close to the cell membrane, as shown by median values of Pearson’s correlation coefficient (0.61, IQR 0.54–0.71) and Mander´s colocalization coefficients for the cell membrane marker and vitronectin (M_G_ 0.86, IQR 0.79–0.94; M_R_ 0.84, IQR 0.76–0.90).

**Fig 5:**
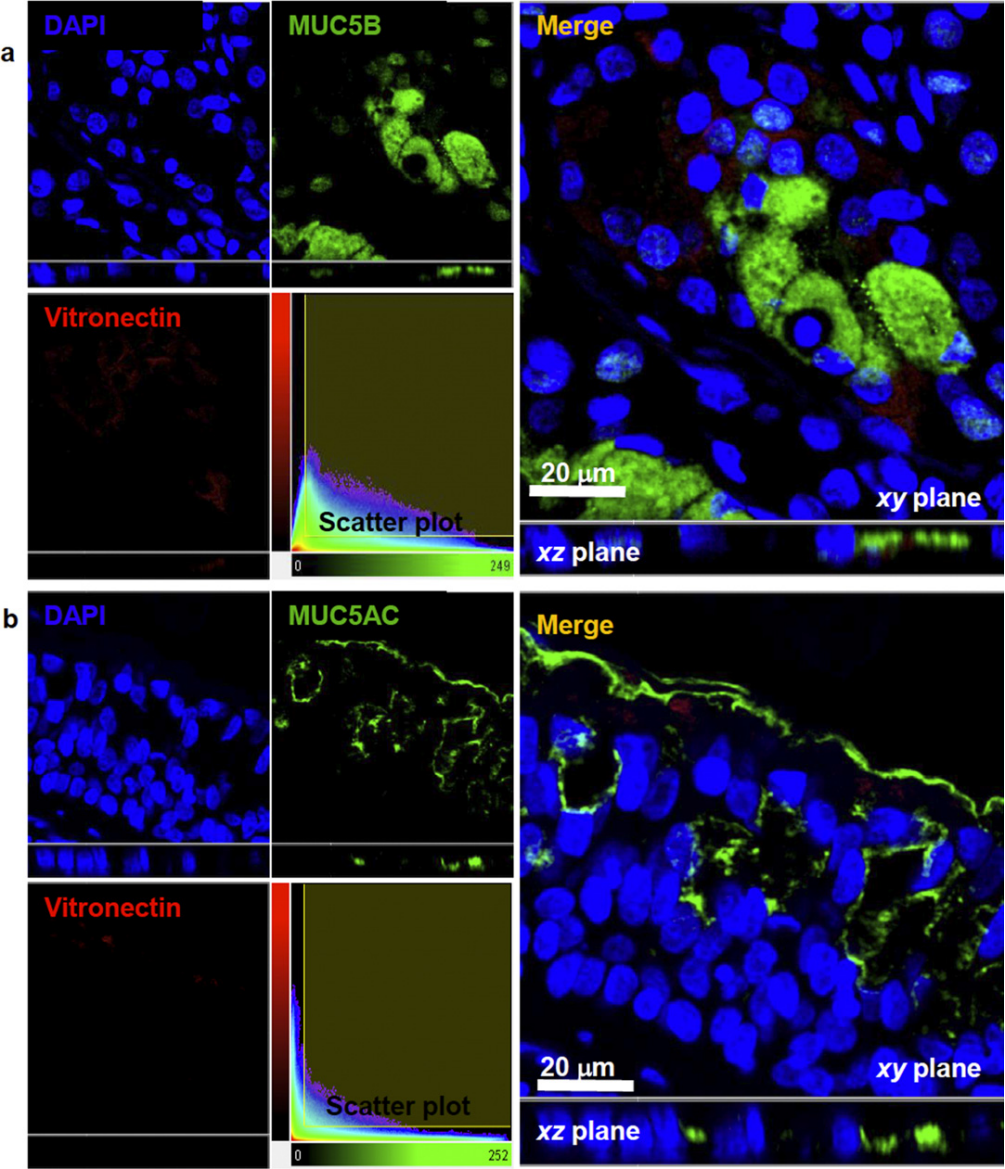
Colocalization of MUC5B or MUC5AC and vitronectin. Confocal images showing localization of MUC5B (**a**) and MUC5AC (**b**) (green fluorescence), as markers of mucous cells in submucosal glands and epithelial goblet cells, respectively and vitronectin (red fluorescence). Overlay of green and red channels showed that vitronectin is not present in cells producing MUC5B (R_p_ 0.16, IQR 0.057–0.42) or MUC5AC (R_p_ 0.04; IQR 0.02–0.13). Nuclei were counterstained with DAPI (blue fluorescence). *xy* and xz views are shown to provide sufficient details of spatial distribution in the 3D subcellular space. The colocalization maps (scatter plots) of voxels are shown. MUC5B and MUC5AC emission intensities are plotted on the x-axis, while vitronectin is plotted on the y-axis. R_p_, Pearson´s colocalization coefficient; IQR, interquartile range.

For double-labeling assay to detect whether vitronectin is inside mucous cells, there was a median value for the Pearson´s coefficient (0.16, IQR 0.06–0.42) indicating that vitronectin is expressed to a much lower extent in this kind of epithelial gland cell (Fig. 5a). As vitronectin was also observed in the surface epithelium, as illustrated in the images of double-labeling of the subunit alpha-1 of sodium/potassium ATPase and vitronectin, we performed additional staining to evaluate vitronectin’s presence inside the goblet cells (Fig. 5b). Pearson´s colocalization coefficient of MUC5AC and vitronectin (0.04, IQR 0.02–0.13) and visual examination of images with this molecular marker revealed that vitronectin was predominantly present in the apical zone of the surface epithelium, but not inside the goblet cells.

**Fig 6:**
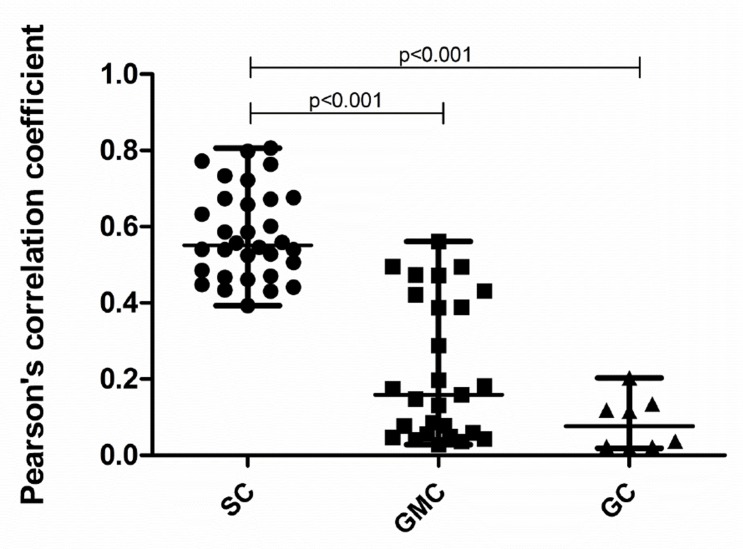
Pearson's Correlation Coefficients for colocalization assays. The plot shows Pearson´s correlation coefficients for the colocalization analysis. For the total sample, medians of R_p_ values for vitronectin and lactoferrin (0.55, IQR 0.47–0.67), vitronectin and MUC5B (0.16, IQR 0.06–0.42), and vitronectin and MUC5AC (0.04, IQR 0.02–0.13 indicate that colocalization was only significant for the marker of serous cell and vitronectin. The Kruskall-Wallis H Test was used for calculating statistical differences. SC, serous cells; GMC, glandular mucous cells; GC, goblet cells.

Pearson’s correlation coefficients relating vitronectin expression in serous cells (SC), glandular mucous cells (GMC) and goblet cells (GC) are plotted in Fig. 6. The values for the inter-quartile range (IQR) are also listed in the figure legend. Median values for Pearson´s and Mander´s coefficients for each experiment and the corresponding groups of subjects from whom the tissues were obtained are presented in Table 2.

**Table 2:** Pearson’s correlation coefficient and Mander’s overlap colocalization coefficients of quantitative confocal analysis.

Coefficients	Normal	Asthmatic	COPD	P
**Cell membrane marker and vitronectin (R_p_)**	0.54 (0.44–0.59)	0.71 (0.62–0.80)	0.67 (0.61–0.75)	0.0471^a^
**Cell membrane marker (M_x_)** ^b^	0.79 (0.71–0.85)	0.87 (0.80–0.93)	0.95 (0.88–0.96)	0.0830
**Vitronectin (M_y_)** ^b^	0.76 (0.66–0.80)	0.90 (0.87–0.94)	0.85 (0.84–0.97)	0.0471 ^a^
**Lactoferrin and vitronectin (R_p_)**	0.72 (0.67–0.78)	0.47 (0.44–0.53)	0.55 (0.50–0.59)	< 0.0001 ^a^
**Lactoferrin (M_x_)** ^c^	0.83 (0.77–0.88)	0.73 (0.62–0.78)	0.63 (0.59–0.68)	0.0017 ^a^
**Vitronectin (M_y_)** ^c^	0.96 (0.93–0.97)	0.92 (0.90–0.93)	0.90 (0.85–0.93)	0.0040 ^a^
**MUC5B and vitronectin (R_p_)**	0.40 (0.17–0.48)	0.08 (0.05–0.16)	0.05 (0.03–0.06)	0.0018 ^a^
**MUC5B (M_x_)** ^d^	0.54 (0.18–0.62)	0.11 (0.06–0.23)	0.14 (0.10–0.17)	0.0031 ^a^
**Vitronectin (M_y_)** ^d^	0.54 (0.44–0.59)	0.71 (0.62–0.80)	0.67 (0.61–0.75)	< 0.0001 ^a^
**MUC5AC and vitronectin (R_p_)**	0.79 (0.71–0.85)	0.87 (0.80–0.93)	0.95 (0.88–0.96)	0.4724
**MUC5AC (M_x_)** ^e^	0.76 (0.66–0.80)	0.90 (0.87–0.94)	0.85 (0.84–0.97)	0.1910
**Vitronectin (M_y_)** ^e^	0.72 (0.67–0.78)	0.47 (0.44–0.53)	0.55 (0.50–0.59)	0.0550

Values are expressed as medians (interquartile ranges). Rp, Pearson’s correlation coefficient; Mx, Mander’s colocalization coefficient for channel 1; My, Mander’s colocalization coefficient for channel 2.

^a^ Statistically significant values by Kruskall-Wallis H test.

^b,c,d,e^ Mander's overlap coefficients for colocalization analysis of cell membrane marker/vitronectin (b), lactoferrina/vitronectin (c), MUC5B/vitronectin (d), and MUC5AC/vitronectin (e).

### Vitronectin expression in asthma and COPD

**Fig 7:**
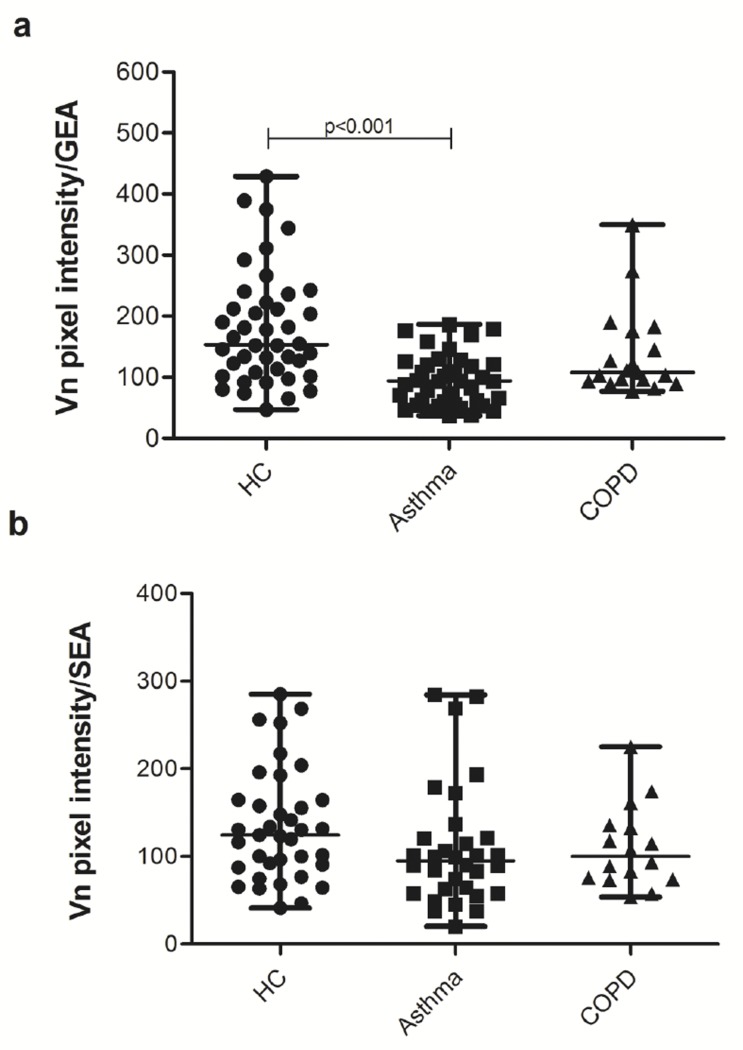
Vitronectin expression in human bronchial tissues. Expression in submucosal glands (**a**), and in respiratory surface epithelium (**b**). The measurements included 7 healthy controls (HC), 6 asthmatic and 4 COPD subjects. For submucosal glands 42 control, 41 asthmatic and 19 COPD acinar structures were evaluated; whereas for respiratory surface epithelium 37 control, 32 asthmatic and 16 COPD tissue structures were assessed. GEA, glandular epithelial area; SEA, surface epithelium area.

As mentioned above, stereological analysis of immunohistochemical images from cadaveric tissues showed that vitronectin expression was significantly lower in asthmatic and COPD subjects when compared with the control group. To further quantify the difference we measured pixel intensities of vitronectin per evaluated area of submucosal glands or surface epithelium (with immunofluorescent labeling of vitronectin), on images from 2D optical sections obtained by confocal microscopy of bronchial tissue from lung donors. A total of 109 images were evaluated (average of six per subject). Median values of measurements were obtained for each subject group. Comparisons of results among the three subject groups are shown in Fig. 7. In bronchial submucosal glands, vitronectin expression was lower in asthmatic and COPD individuals when compared with healthy controls. Even though, this difference was only statistically significant for asthmatic individuals versus HC (p<0.0001) (Fig. 7a). The lack of statistical difference between the COPD and control groups could be due to the small sample size of the COPD group (n = 4). There was no difference in the vitronectin expression in the surface epithelium among the groups (Fig. 7b).

### Vitronectin coding RNA is present in bronchial surface epithelial cells

**Fig 8:**
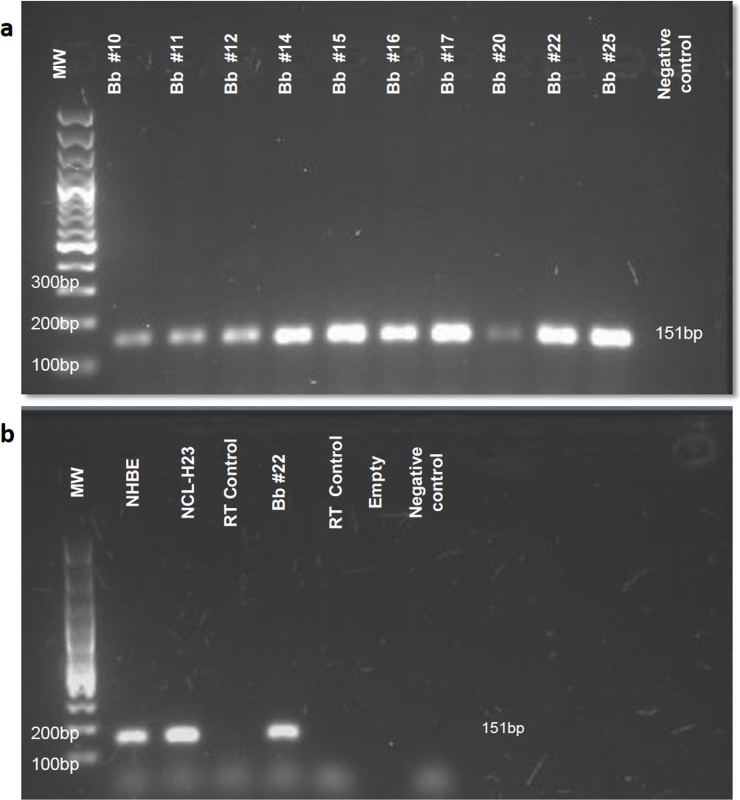
(a) Agarose gel electrophoresis of the amplification product for vitronectin cDNA from bronchial brushings. The amplified fragment corresponds to a band size of 151bp. MW, molecular weight marker, Bb, bronchial brushing, and NC, negative control or “non-template control” of qPCR. The number accompanying bronchial brushings corresponds to the number assigned to different patients. **(b) Agarose gel electrophoresis of the amplification product for vitronectin cDNA from cultured cells**. Molecular weight marker (MW), normal human bronchial epithelial cells (NHBE), cells from adenocarcinoma of epithelial origin (NCL-H23), bronchial brushing (Bb), extraction and retro-transcription control (RT control) or “no reverse-transcriptase control”, and negative control (NC).

In order to obtain further evidence indicating production of vitronectin by resident cells of the bronchial epithelial layer, vitronectin RNA expression was measured in bronchial brushings, as well as in cell lines of normal human tracheal/bronchial epithelium (primary cells) and lung adenocarcinoma of epithelial origin. A total of 12 bronchial brushings from subjects who underwent diagnostic bronchoscopy were evaluated, with vitronectin coding RNA detected in all of them (Fig. 8a). Vitronectin RNA expression in samples of patients with lung cancer or lung infection was not different compared to the control group (p = 0.281, 95% CI, 0.634–1.159). Transcription of *VTN* gene was also demonstrated in cultured NHBE and NCL-H23 cells (Fig. 8b). It should be pointed out that the results of our 2-D cell culture may not be the same as those from more physiological 3-D cell cultures.

### The vitronectin isoforms (65 kDa and 75 kDa) are expressed differentially in bronchial brushing samples

**Fig 9:**
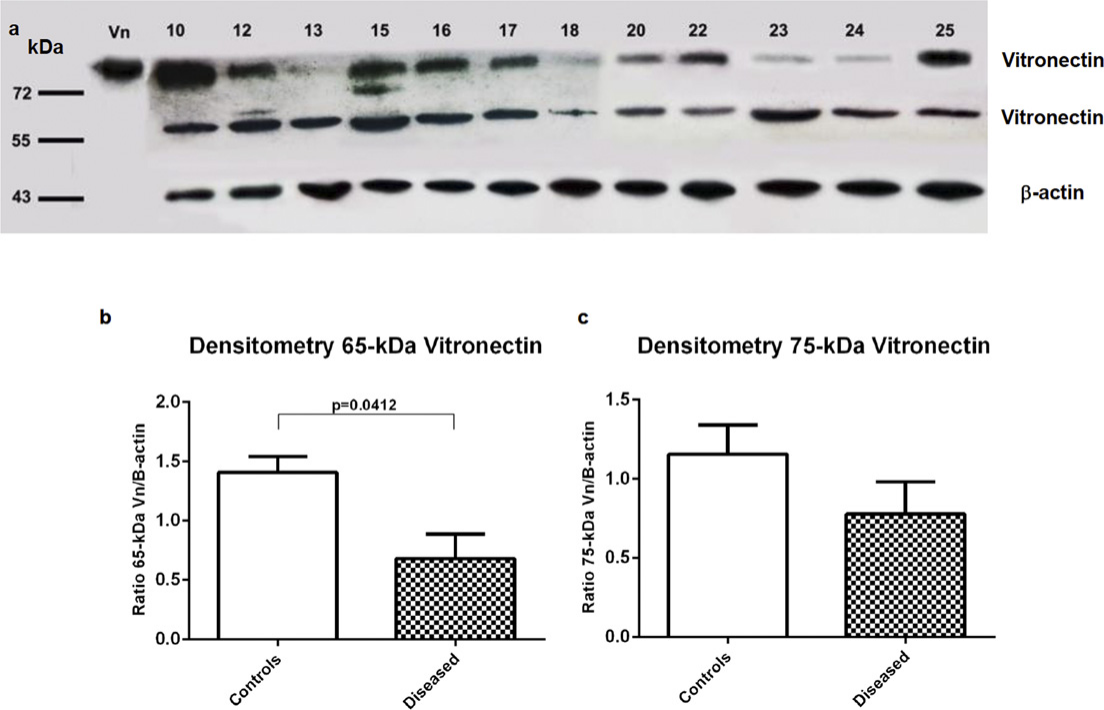
Western analysis of vitronectin isoforms expression in bronchial surface epithelium from bronchial brushings. **(a)** Immunoblots of vitronectin expression in control individuals (number 10, 15 and 17) and diseased subjects (numbers 12, 13, 16, 18, 20, 22, 23, 24 and 25). 65- and 75-kDa vitronectin isoforms are presents in all subjects. β-actin was used as a control for protein loading. **(b-c) Quantification by densitometry of 65- and 75-kDa vitronectin isoforms.** 65-kDa vitronectin expression was lower in diseased subjects when compared with control individuals (p = 0.0412). The vitronectin/β-actin ratio is represented on the y axes for controls and diseased subjects. Error bars represent the standard error of means.

To quantitatively evaluate the expression of vitronectin isoforms in bronchial surface epithelial cells, western-blot analysis was carried out in a total of 12 bronchial brushings samples. The vitronectin isoforms were observed in both controls (with normal bronchoscopic, histological and microbiological findings) and diseased subjects (Fig. 9a). Densitometry analysis of vitronectin isoforms on western blots showed that the diseased subjects had decreased expression for the 65-kDa vitronectin isoform when compared with the control group (p = 0.0412) (Fig. 9b). There was no difference in the 75-kDa isoform expression between the two groups (p = 0.1702) (Fig. 9c).

## Discussion

The present study has shown that the glycoprotein vitronectin is expressed by submucosal serous acinar cells in the bronchi of control subjects as well as of asthmatic and COPD individuals, although the expression is significantly lower in the diseased group. The glycoprotein is also expressed by tracheobronchial surface epithelium, especially in the apical zone. This is the first report of vitronectin expression by resident cells in human airways.

Vitronectin production by hepatocytes [5,51], retinal pigment epithelium [52–54] and the urothelium of lower urinary tract [55] has been reported. Hepatocytes are generally recognized as the primary source of vitronectin in plasma and ECM. The present finding indicates that besides the plasma source, vitronectin is also locally produced in the airways. The reason for the need of a local supply of vitronectin is not clear, but one could speculate that the supply of vitronectin via circulation may not meet all the local needs.

The airway epithelial layer is able to serve as a physical barrier to inhaled pathogens due to the intercellular epithelial junctions and the mucus layer. In addition, pathogen recognition and secretion of cytokines, chemokines and antimicrobial peptides by the epithelium, in concert with the ciliary function, constitutes an important part of the innate immune response of the airways. Production of vitronectin by resident cells of the airway epithelial and submucosal layer is likely an integral part of the airway innate immune response.

Vitronectin has three heparin-binding domains, located at the protein’s carboxi-terminal region that are responsible for the interaction with diverse glycosaminoglycans (GAGs), as well as for the inhibition of complement activation [56,57]. The heparin-binding domains of vitronectin not only interacts with ECM and serum proteins, but also with microbial components [17–19,21,22]. These domains exhibit amphipathic properties which facilitate interaction of vitronectin with GAGs and negatively charged microbial membranes, leading to microbial membrane permeabilization and killing of the microbes. [15,16].

The antimicrobial and immune-cell-recruiting abilities of vitronectin, through its RGD motif [4,58,59], make the glycoprotein an ideal player in the immune system. Another property of vitronectin that makes it indispensable for maintaining airway health is its inhibitory ability of MAC formation that confers protection to host cells during infection [6,60]. One can envisage that without such protection extensive damage to the airway cells would accumulate during recurring infections, and in the long run this could lead to irreversible scar tissue formation and airway remodeling due to repeated cycles of injury and repair. The protective effect of vitronectin appears to come with a price, and that is, the glycoprotein also plays a role in promoting microbial adhesion and internalization [19].

It is interesting that vitronectin expression is reduced in asthma (and to a lesser extent, in COPD), even though the underlying mechanism is not clear. A reduced level of vitronectin in the airways may compromise the host’s ability to fight infection, because of the intrinsic antimicrobial [15,16] and immune-cell-recruiting properties of the glycoprotein. However, the reduced level may also lessen the chance of microbial invasion, because of the ability of vitronectin to tether bacteria to the integrin receptor on host epithelial cells that promotes internalization of pathogens [22]. Whether there is any beneficial effect in asthma and COPD to have a reduced level of vitronectin expression is difficult to assess without further information. On the other hand, the detrimental effect of low vitronectin concentration in the airways of asthmatic and COPD subjects appears to be clear. Without the inhibitory effect of vitronectin on MAC formation, self-inflicted assault of airway cells with each episode of colonization by a pathogen may lead to exaggerated immune response and injury and subsequent tissue repair. Over time this could lead to airway remodeling, a hallmark of persistent asthma [61]. The underlying mechanism for the reduction in vitronectin production and the exact role of vitronectin in airway remodeling need to be further investigated.

Many studies of vitronectin gene expression *in vivo*, both in murine and human tissues, have suggested that this glycoprotein is regulated in a tissue-specific manner [62,63,54]. It has been demonstrated that hepatic vitronectin gene expression is up-regulated in acute and chronic inflammation [63]. Conversely, when vitronectin coding RNA levels were evaluated in surface epithelium from bronchial brushings, there was no difference between the control and the diseased groups (with lung cancer or infection). However, the 65-kDa isoform expression was lower in the diseased subjects. This isoform is believe to be generated from the 75-kDa isoform, which depends on the presence of threonine or methionine at position 381, as described by Fink et al [64]. The presence of threonine rather than methionine at position 381 was proposed to be responsible for the susceptibility for vitronectin to be cleaved at Arg^379^-Ala^380^ [60]. Studies performed by Tschopp *et al* have suggested that a two-chain isoform (65 + 10 kDa) could be more efficient to inhibit the MAC formation [57]. Thus, a decrease of the 65-kDa vitronectin isoform could increase the susceptibility to tissue injury by complement activity.

To conclude, vitronectin has been found to be locally produced in the human airways by glandular serous and epithelial cells, and the level of expression of this glycoprotein is reduced in the airways of asthmatic and likely also in COPD subjects. Low levels of vitronectin could contribute to the airway remodeling seen in obstructive airway diseases.

## Supporting Information

S1 Fig Localization of vitronectin in human bronchial epithelium. Image of immunohistochemical labeling for vitronectin (a), Periodic Acid—Schiff (PAS) staining (b), and superposition of both images (c) using Fiji software. As PAS is a specific for mucous cells, it appears that vitronectin expression is confined to the serous component of the airways submucosal glands (arrows), magnification x10.
(TIF)

S1 Methods Supplementary methods. (DOC)

S1 Table Demographics of subjects underwent autopsy at the National Institute of Legal Medicine and Forensic Sciences (Medellín-Colombia). (DOC)

S2 Table Demographics of human lung donors registered at the International Institute for the Advancement of Medicine (Edison, NJ) and James Hogg Research Centre Biobank (Vancouver, BC). (DOC)

S3 Table Demographics and bronchoscopic findings of the study population used for RNA extraction (Clínica Cardio VID, Medellín, Colombia). (DOC)

S4 Table Antibodies used for immunohistochemistry and immunofluorescence analysis. (DOC)

S5 Table Primer sequences, melting temperatures and sizes of the PCR products used in RNA analysis. (DOC)
