# Supplementary figures and images for: Vitronectin Expression in the Airways of Subjects with Asthma and Chronic Obstructive Pulmonary Disease
Source: PLoS One. 2015 Mar 13;10(3):e0119717. doi: 10.1371/journal.pone.0119717 (PMC4358944; doi:10.1371/journal.pone.0119717)

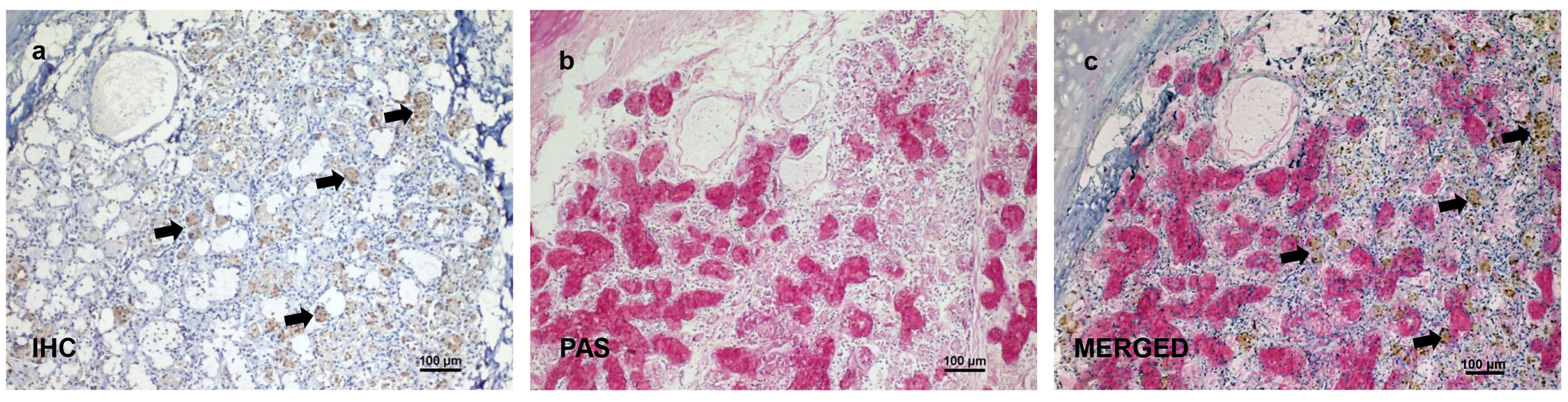

Supplement: S1 Fig — Image of immunohistochemical labeling for vitronectin (a), Periodic Acid—Schiff (PAS) staining (b), and superposition of both images (c) using Fiji software. As PAS is a specific for mucous cells, it appears that vitronectin expression is confined to the serous component of the airways submucosal glands (arrows), magnification x10. (TIF) [file pone.0119717.s001.tif]
