# Supplementary material for: Vitronectin Expression in the Airways of Subjects with Asthma and Chronic Obstructive Pulmonary Disease
Source: PLoS One. 2015 Mar 13;10(3):e0119717. doi: 10.1371/journal.pone.0119717 (PMC4358944; doi:10.1371/journal.pone.0119717)
Supplement: S1 Methods — (DOC) [file pone.0119717.s002.doc]

**S1 Methods. Supplementary Methods**

*Tissue samples*

To demonstrate the presence of vitronectin RNA and protein in bronchial surface epithelium, brushings from 12 patients who underwent diagnostic bronchoscopy at Clínica Cardio VID (Pulmonology Service, Medellín, Colombia) were sampled (Online supplement, Table 3). Bronchoscopy was done under mild sedation with midazolam, and anesthesia of the vocal cords and bronchial airways with topical lidocaine. The video bronchoscope (Olympus, CV-100), with a working channel of 2 mm in diameter, was positioned proximal to the opening of a lobar bronchus without apparent morphological abnormalities. Surface epithelial cells were obtained by gently gliding a disposable cytology brush (Olympus) back and forth on the bronchial wall, five to ten times in the same general area. Later, the head of the brush was cut off, and immersed into 800 µL of RNAlater® (Life Technologies^TM^, Carlsbad, CA, USA) solution. Samples were stored at -80°C until processing.

*Immunohistochemical staining*

The cadaveric tissue sections were deparaffinized with xylene (Sigma-Aldrich®; St. Louis, MO, USA) and rehydrated in downgraded isopropanol solutions from absolute alcohol (Mallinckrodt® Chemicals; St. Louis, MO, USA). All washes were performed with 0.025% Triton X-100 prepared in 1X tris buffered saline (TBS), pH 7.4. In order to quench endogenous peroxidase, the sections were treated with 3% hydrogen peroxide (Sigma-Aldrich®; St. Louis, MO, USA) prepared in 1X TBS. The heat-induced epitope retrieval was performed with 10mM sodium citrate, pH 6.0 (Dako, Denmark). Nonspecific binding of primary antibody was prevented by treatment with 1% normal goat serum (Sigma-Aldrich®; St. Louis, MO, USA) at room temperature for 1 hour. Primary antibody was used in a dilution of 1:400 in 1% bovine serum albumin (BSA) (Sigma-Aldrich®; St. Louis, MO, USA) in 1X TBS. Incubation was performed for 3 hours at room temperature. Later on, sections were incubated with secondary antibody diluted 1:150 in 1% BSA in TBS, at room temperature for 1 hour. Staining pattern was developed with a freshly made solution of 3,3’diaminobenzidine tetrahydrocloride (amresco®; Solon, OH, USA). Sections were counterstained with hematoxylin (Thermo Fisher Scientific; Suwanee, GA, USA), cleared with ammoniacal alcohol (Novalab; Medellín, Colombia), dehydrated in upgraded isopropanol and mounted in shandon consul-mount (Thermo Fisher Scientific; Suwanee, GA, USA). As a negative control, the primary antibody was substituted with 1% BSA in TBS. The specificity of primary antibody to human vitronectin was tested and confirmed by the supplier with immunoblotting.

*Immunofluorescence staining*

Donor airway sections (7 μm thickness) were deparaffinized and rehydrated in downgraded ethanol solutions. To reduce fixative-induced fluorescence, sections were treated with 0.1 g/mL of sodium borohydrate (Sigma-Aldrich®; St. Louis, MO, USA) in phosphate buffered saline (PBS) for five minutes [45]. Antigens were retrieved by autoclaving the sections in citrate buffer target retrieval solution (Dako, Denmark) for 15 minutes at 120°C and 30 psi. Non-specific binding was blocked by incubating sections with 5% normal goat serum (NGS) (Life Technologies^TM^; Grand Island, NY, USA) at room temperature for 20 minutes. Slides were co-incubated overnight at 4°C with antibodies against vitronectin (1:100) and either a plasma membrane marker alpha-1 sodium/potassium ATPase antibody (1:200), lactoferrin (1:100), MUC5B (1:75) or MUC5AC (1:400). Antibodies were diluted in 2.5% NGS. After removing the primary antibodies and washing the sections with PBS, secondary antibodies (dilution 1/100) were added and incubated for 2 hours at room temperature. Sections were washed again before dehydration, and mounted in Vectashield Hardset mounting medium with DAPI (Vector Laboratories; Burlingame, CA, USA). As a negative control, the primary antibody was substituted with 2.5% NGS in PBS. The specificity of the primary antibodies was tested and confirmed by the suppliers with immunoblotting, flow cytometry, immunohistochemistry and immunofluorescence.

*Western-blot analysis*

Western-blot analysis were used to quantitatively assess vitronectin expression in bronchial brushing samples. Equal concentration of solution protein mixed with 20µL of sample buffer and 5µl of reducing agent was heated at 95°C for 5 minutes. Then, samples were loaded on 8% polyacrylamide gel. Protein electrophoresis was carried out at 80 volts, for 30 minutes at 22°C, and then increased the voltage to 120 for 60 minutes at 22°C. Sample proteins were transferred (45volts, 1 hour at 4°C) to a 0.2µm PVDF membrane (Bio-Rad, Hercules, CA, USA) in 5% isopropanol transfer buffer. After, the membranes were blocked with 5% non-fat milk in TBS with 0.1% tween-20 (TBST 1X) for 60 minutes. The membranes were incubated with mouse monoclonal anti-human vitronectin antibody (1:600) (clone VIT-2, isotype IgM, code V7881) (Sigma-Aldrich®; St. Louis, MO, USA) and monoclonal anti β- actin antibody (1:5000) (Clone AC-74 code A2228) (Sigma-Aldrich®; St. Louis, MO, USA), at 4°C overnight. Detection was performed using as secondary antibodies anti-mouse IgM peroxidase conjugated antibody produced in goat (μ-chain specific, code A8786) (Sigma-Aldrich®; St. Louis, MO, USA) and anti-mouse IgG peroxidase conjugated antibody produced in rabbit (whole molecule, code A9044) (Sigma-Aldrich®; St. Louis, MO, USA), respectively. Then, an enhanced chemiluminescent reagent system was used (Bio-Rad, Hercules, CA, USA).

To assess the blot quantitatively, the PVDF membrane were revealed using X-ray film (Amersham Hyperfilm ECL) (GE Healthcare Bio-Sciences Corp., Piscataway, NJ), maintaining exposure within the linear range of detection. After X-ray film development, a digital image were created by scanning the films for further computer analysis using ImageJ 1.48 software.
