# Supplementary material for: Vitronectin Expression in the Airways of Subjects with Asthma and Chronic Obstructive Pulmonary Disease
Source: PLoS One. 2015 Mar 13;10(3):e0119717. doi: 10.1371/journal.pone.0119717 (PMC4358944; doi:10.1371/journal.pone.0119717)
Supplement: S1 Table — (DOC) [file pone.0119717.s003.doc]

**S1 Table.** **Demographics of subjects underwent autopsy at the National Institute of Legal Medicine and Forensic Sciences (Medellín-Colombia).**

| **Subject code** | **Gender** | **Age (years)** | **Disease status** | **Cause of death** |
| --- | --- | --- | --- | --- |
| 1 | Male | 25 | Healthy control | Brain injury caused by gunshot wounds |
| 2 | Male | 14 | Healthy control | Gunshot wounds |
| 3 | Male | 37 | Healthy control | Gunshot wounds |
| 4 | Male | 54 | Healthy control | Stab wound |
| 5 | Male | 38 | Healthy control | Gunshot wounds |
| 6 | Male | 38 | Healthy control | Traffic accident |
| 7 | Male | 25 | Healthy control | Mechanical suffocation |
| 8 | Male | 23 | Healthy control | Gunshot wounds |
| 9 | Male | 27 | Healthy control | Gunshot wounds |
| 10 | Male | 38 | Healthy control | Gunshot wounds |
| 11 | Male | 51 | Healthy control | Gunshot wounds |
| 12 | Female | 28 | Healthy control | Gunshot wounds |
| 13 | Male | 29 | Healthy control | Gunshot wounds |
| 14 | Male | 19 | Asthma | Brain injury after a fall |
| 15 | Male | 36 | Asthma | Traumatic brain injury |
| 16 | Male | 54 | Asthma | Cardiogenic shock |
| 17 | Male | 33 | Asthma | Not registered |
| 18 | Male | 27 | Asthma | Gunshot wounds |
| 19 | Male | 35 | Asthma | Traffic accident |
| 20 | Male | 24 | Asthma | Gunshot wounds |
| 21 | Female | 38 | Asthma | Cardiogenic shock due to ventricular hypertrophy |
| 22 | Male | 43 | COPD | Stab wounds |
| 23 | Male | 44 | COPD | Brain injury after a traffic accident |
| 24 | Male | 40 | COPD | Gut volvulus |
| 25 | Female | 76 | COPD | Septic shock secondary to soft-tissue injury |
| 26 | Male | 69 | COPD | Gunshot wounds |
| 27 | Male | 57 | COPD | Acute myocardial infarction |
| 28 | Male | 51 | COPD | Not registered |
| 29 | Male | 64 | COPD | Gastrointestinal bleeding due to liver cirrhosis |
| 30 | Female | 92 | COPD | Hospital-acquired pneumonia and sepsis |
| 31 | Male | 71 | COPD | Pneumonia secondary to traumatic brain injury |
