# Supplementary material for: Vitronectin Expression in the Airways of Subjects with Asthma and Chronic Obstructive Pulmonary Disease
Source: PLoS One. 2015 Mar 13;10(3):e0119717. doi: 10.1371/journal.pone.0119717 (PMC4358944; doi:10.1371/journal.pone.0119717)
Supplement: S2 Table — (DOC) [file pone.0119717.s004.doc]

**S2 Table.** **Demographics of human lung donors registered at the International Institute for the Advancement of Medicine (Edison, NJ) and James Hogg Research Centre Biobank (Vancouver, BC).**

| **Patient code** | **Gender** | **Age (years)** | **Disease status** | **Cause of death** |
| --- | --- | --- | --- | --- |
| 1 | Male | 51 | Healthy control | Unknown |
| 2 | Male | 62 | Healthy control | Unknown |
| 3 | Male | 36 | Healthy control | Unknown |
| 4 | Male | 56 | Healthy control | Unknown |
| 5 | Male | 52 | Healthy control | Unknown |
| 6 | Male | 43 | Healthy control | Unknown |
| 7 | Male | 59 | Healthy control | Unknown |
| 8 | Female | 8 | Asthma | Fatal asthma |
| 9 | Female | 26 | Asthma | Fatal asthma |
| 10 | Female | 15 | Asthma | Fatal asthma |
| 11 | Male | 36 | Asthma | Head trauma |
| 12 | Male | 23 | Asthma | Fatal asthma |
| 13 | Male | 10 | Asthma | Fatal asthma |
| 14 | Female | 52 | COPD | Unknown |
| 15 | Female | 65 | COPD | Unknown |
| 16 | Female | 56 | COPD | Unknown |
| 17 | Male | 58 | COPD | Unknown |
