# Supplementary material for: Vitronectin Expression in the Airways of Subjects with Asthma and Chronic Obstructive Pulmonary Disease
Source: PLoS One. 2015 Mar 13;10(3):e0119717. doi: 10.1371/journal.pone.0119717 (PMC4358944; doi:10.1371/journal.pone.0119717)
Supplement: S3 Table — (DOC) [file pone.0119717.s005.doc]

**S3 Table. Demographics and bronchoscopic findings of the study population used for RNA extraction (Clínica Cardio VID, Medellín, Colombia)**.

| **Patient N°** | **Gender** | **Age (years)** | **Lung transplant recipient** | **Diagnosis** |
| --- | --- | --- | --- | --- |
| 10 | Female | 73 | No | Healthy control bronchoscopic and microbiological findings |
| 12 | Male | 47 | Yes | Negative for lung transplant rejection |
| 13 | Male | 51 | Yes | Mainstem bronchial stenosis |
| 15 | Male | 64 | No | Healthy control bronchoscopic and microbiological findings |
| 16 | Male | 74 | No | Lung adenocarcinoma |
| 17 | Male | 25 | No | Healthy control bronchoscopic and microbiological findings |
| 18 | Female | 62 | No | Pulmonary infiltrates |
| 20 | Female | 45 | No | Lung cancer |
| 22 | Male | 76 | No | Non-small cell lung carcinoma (NSCLC) without metastasis |
| 23 | Female | 58 | Yes | Pulmonary aspergillosis |
| 24 | Male | 69 | No | Bacterial pneumonia |
| 25 | Male | 57 | No | Community-acquired pneumonia |
