# Supplementary material for: Vitronectin Expression in the Airways of Subjects with Asthma and Chronic Obstructive Pulmonary Disease
Source: PLoS One. 2015 Mar 13;10(3):e0119717. doi: 10.1371/journal.pone.0119717 (PMC4358944; doi:10.1371/journal.pone.0119717)
Supplement: S4 Table — (DOC) [file pone.0119717.s006.doc]

**S4 Table.** **Antibodies used for immunohistochemistry and immunofluorescence analysis.**

| **Target** | **Antibody - Clone** | **Isotype** | **Immunogen** | **Purity** | **Source** |
| --- | --- | --- | --- | --- | --- |
| Vitronectin | Mouse monoclonal [VIT-2] to human vitronectin | IgM | Purified human plasma  vitronectin | Ascites fluid | Sigma |
| Mouse IgM | Goat anti-mouse IgM (μ-chain specific) conjugated to horseradish peroxidase |  | Purified mouse IgM | Affinity isolated antibody | Sigma |
| Vitronectin | Rabbit monoclonal [EP873Y] to vitronectin | IgG1 | Synthetic peptide corresponding to residues near the V10 subunit of human vitronectin | Tissue culture supernatant | Abcam |
| Rabbit IgG | Goat anti-rabbit IgG antibody, highly cross-adsorbed and labeled with Alexa Fluor® 594 |  |  | Affinity-purified antibodies that react with IgG heavy chains and all classes of immunoglobulin light chains from mouse | Life Technologies |
| Cell membrane marker  (Na^+^/K^+^-ATPase) | Mouse monoclonal [464.6] to Na^+^/K^+^-ATPase | IgG1 | Full length native protein purified from rabbit renal outer medulla | IgG fraction | Abcam |
| Serous cell marker  (Lactoferrin) | Mouse monoclonal (2B8) to lactoferrin | IgG1 | Full length human native protein (purified) | Protein G purified | Abcam |
| Submucosal glands mucous cell  (MUC5B) | Mouse monoclonal [8C11] to human MUC5B | IgG2a Kappa | Mucin 5B partial (XP-039877, 4186 - ~ 4296aa) recombinant with glutathione S-transferase tag | Ascites fluid | Sigma |
| Goblet cell  (MUC5AC) | Mouse monoclonal [45M1] to mucin 5AC | IgG1 | M1 mucin preparation from the fluid of an ovarian mucinous cyst | IgG fraction | Abcam |
| Mouse IgG | Goat anti-mouse IgG antibody, highly cross-adsorbed and labeled with Alexa Fluor® 488 |  |  | Affinity-purified antibodies that react with IgG heavy chains and all classes of immunoglobulin light chains from mouse | Life Technologies |
