# Supplementary material for: Vitronectin Expression in the Airways of Subjects with Asthma and Chronic Obstructive Pulmonary Disease
Source: PLoS One. 2015 Mar 13;10(3):e0119717. doi: 10.1371/journal.pone.0119717 (PMC4358944; doi:10.1371/journal.pone.0119717)
Supplement: S5 Table — (DOC) [file pone.0119717.s007.doc]

**S5 Table.** **Primer sequences, melting temperatures and sizes of the PCR products used in RNA analysis.**

| **Marker** | **Target sequences** | **Primer sequence 5´→ 3´** | **Annealing temperature**  **(°C)** | **Product size**  **(bp)** |
| --- | --- | --- | --- | --- |
| GADPH | NM 002046.4 | **Forward** GAA GGT CGG AGT CAA CGG ATT  **Reverse** GCC ATG GGT GGA ATC ATA TTG GA | 60.07  60.75 | 151pb |
| Vitronectin | NM 000638.3 | **Forward** GGC TGT CCT TGT TCT CCA GTG  **Reverse** GTG CGA AGA TTG ACT CGG TAG T | 60.88  60.42 | 151pb |
